# Silane Heat Treatment Could Eliminate the Hydrofluoric Acid Etching of Lithium Disilicate Overlays: A Four-Year Follow-Up

**DOI:** 10.1155/2021/9961621

**Published:** 2021-06-22

**Authors:** Spartak Spasov Yanakiev, Mirela Borislavova Marinova-Takorova

**Affiliations:** ^1^Medical College, Medical University of Sofia, Yordanka Filaretova Str. No. 3, Sofia, Bulgaria; ^2^Department of Conservative Dentistry, Faculty of Dental Medicine, Medical University of Sofia, Georgi Sofiiski 3 Str., Sofia, Bulgaria

## Abstract

A four-year follow-up of a novel silane heat treatment method for bonding lithium disilicate overlays to tooth structures without hydrofluoric acid etching of the ceramic surface is presented in this case report. Silane heat treatment modifies the silane layer and thus enhances resin ceramic bond strength without hydrofluoric acid etching. The standard ceramic preparation technique prior to bonding silicate ceramics to tooth structure is hydrofluoric acid etching and applying a silane coupling agent, followed by dental adhesive. In this case, the micromechanical roughening of the ceramic surface was performed by air abrasion with Al_2_O_3_. Silane heat treatment with constant 120°C airflow, applied for 60 sec, followed by dental adhesive application enhanced the resin-ceramic bond strength. After a four-year follow-up, the restorations' clinical appearance could be defined as excellent/very good according to the FDI clinical criteria for the evaluation of direct and indirect restorations. This clinical result supports many in vitro studies regarding the resin-ceramic bond strength and durability obtained through postsilanization heat treatment.

## 1. Introduction

Lithium disilicate (LiS_2_) is one of the most widely applied metal-free materials due to its high aesthetics, adequate mechanical properties, and favorable bonding strength, ensuring functional durability and high short- and medium-term survival rates [1–4]. It can be used for both tooth- and implant-supported restorations, including single crowns, bridges, anterior veneers, posterior inlays, onlays, and overlays [5]. In cases when single restorations like inlays, onlays, and partial crowns are needed, they present advantages such as high fracture resistance, low wear and abrasive potential, biocompatibility, adhesive bonding, and strength which make them preferable in cases with significant loss of tooth structure and abrasion or when occlusal correction is needed [5]. Bonded restorations with full occlusal coverage preserve tooth structure compared to full-coverage crowns and increase the fracture resistance of endodontically treated teeth compared to direct MOD restorations [6]. LiS_2_ has shown promising clinical results for posterior endodontically treated teeth with or without fiber posts [7]. These restorations' success depends on many factors—tooth preparation, presence of enamel, bonding procedures, and material selection [8, 9].

The adequate adhesive bonding procedure for glass-ceramics needs proper preparation of the inner surface of the restoration. The significant adhesive strength is due to both micromechanical and chemical bonding mechanisms. The surface microirregularities for the micromechanical interlocking between the ceramic and the cement could be made by acid etching, sandblasting, or diamond bur grinding [5]. The application of a silane coupling agent ensures chemical interaction between the ceramics and the adhesive resin [10].

Hydrofluoric acid (HF) etching of glass-ceramics proved very efficient since it creates a large bonding surface [11, 12]. On the other hand, if it comes in contact with skin, eyes, or mucosa, HF could be harmful to both the patient and the clinician [13, 14] and is acknowledged as a highly hazardous chemical in dentistry [15, 16]. That is why some authors exert efforts on improving the chemical bond between dental ceramics and composite resin provided by the silane coupling agents, thus trying to eliminate the use of HF acid. Silane treatment with heated airflow or in an oven is one of the most frequently used methods [17–20]. The proposed heating temperatures vary from 38 to 100°C. In a recent study, a 120°C heating temperature of the silane was presented [21]. This temperature was the most efficient treatment method, providing the highest bond strength among all heating temperatures. According to some studies, HF's effect in the bonding procedure could be compensated by micromechanical roughening of the ceramic restoration and silane heat treatment to obtain high bond strength [17, 18]. Despite the large number of in vitro studies in the literature, there is still a lack of clinical evidence of these methods' effectiveness in vivo.

This clinical case report describes two ceramic overlays' bonding procedures made from LiS_2_ without HF etching and a 4-year follow-up.

## 2. Case Description

The patient is a 34-year-old male, a smoker with good oral hygiene, who presented into our office for a routine examination. There was secondary caries around a composite resin restoration on tooth 36. The X-ray image of the tooth verified secondary caries on tooth 36 and revealed poor marginal adaptation of the restoration and an inadequate root canal treatment of tooth 37. After discussing the clinical options, the patient signed informed consent—root canal treatment and ceramic overlay restorations of both teeth were planned.

**Figure 1:**
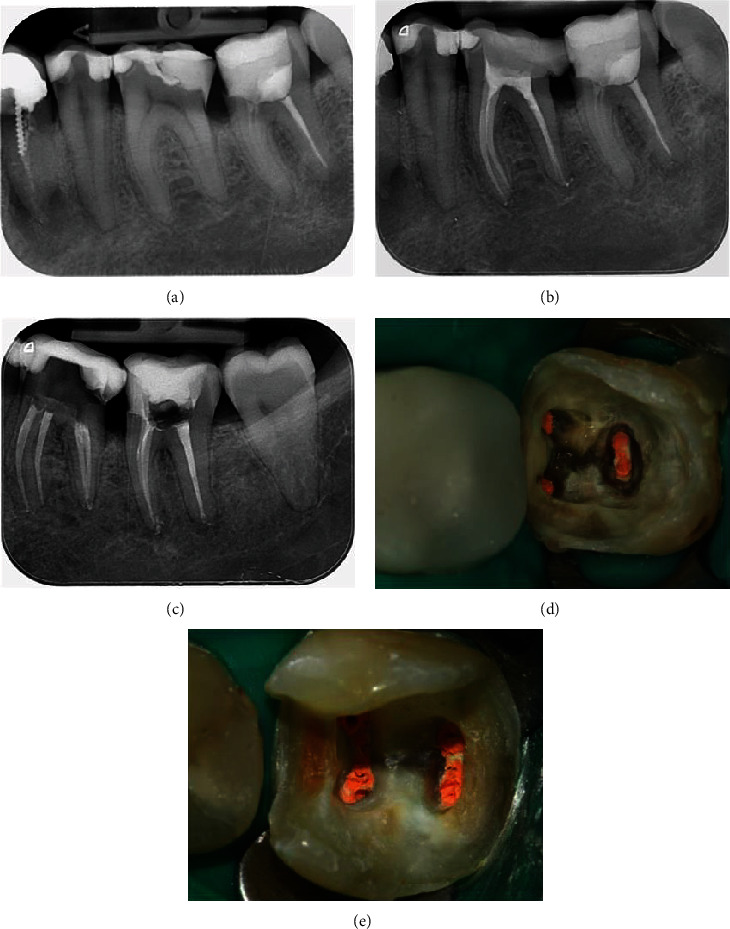
Preoperative images: radiograph images of tooth 36 (a) and 37 (b) prior to endodontic treatment and postendodontic treatment (c) and clinical view of tooth 37 (d) and 36 (e).

The endodontic treatment of the teeth was performed under microscope Zumax OMS2350 (Zumax Medical Co., Ltd., Suzhou New District, China) in several visits one by one. Root canals were shaped with MTwo files, size 25/0.06 (VDW GmbH, Munich, Germany). After copious irrigation with NaOCl solution (Chloraxid 2%, Cerkamed, Stalowa Wola, Poland), the final irrigation with EDTA solution (MD-Cleanser, Meta Biomed Europe GmbH, Mülheim an der Ruhr, Germany) and ethanol 95% (Chemax Pharma, Sofia, Bulgaria) was done before filling the canals with AH plus root canal sealer (Dentsply DeTrey, GmbH, Konstanz, Germany) and taper-matched gutta-percha cone (Sure Dent, Sagimakgol-ro, Jungwon-gu, Seongnam-si, Gyeonggi-do, South Korea) utilizing a single-cone technique (Figure 1).

Tooth preparation for the overlays was performed, and provisional acrylic restorations were placed (Unifast III, GC Corporation, Tokyo, Japan). Full-tray maxillary and mandibular impressions were taken on the next visit using VPS material (Variotime, Heraeus Kulzer GmbH, Hanau, Germany). Two LiS_2_ overlays were fabricated from E.max Press, shade А 3.5 HT (Ivoclar Vivadent, Schaan, Liechtenstein).

**Figure 2:**
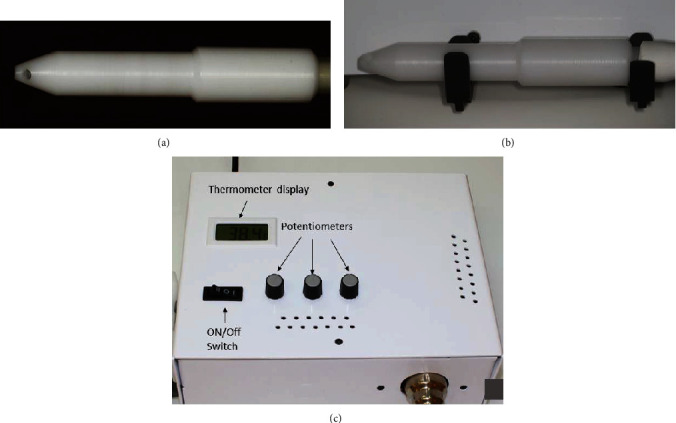
The custom-made air machine used in this study. Autoclavable handpiece made from polyacetate material (placed on the side wall of the device) (a, b). Front panel of the device and basic components (c).

The two ceramic overlays were air abraded with 50 *μ*m Al_2_O_3_ at 1 bar. After the try-in procedures, the inner surfaces of the overlays were cleaned with ethanol and air-dried. A silane coupling agent (Monobond Plus, Ivoclar Vivadent, Schaan, Liechtenstein) was applied for 60 seconds. The key step in the protocol was the silane heat treatment performed with a custom-made hot air machine (Figure 2). The device is equipped with an autoclavable handpiece made from polyacetate material TECAFORM AH POM (Modern Plastics, Connecticut, USA). A thermocouple located in the working end of the handpiece measures the air temperature as near as possible to the treated surface. Temperature is measured to the first decimal place in degrees Celsius. The temperature range supported by the device is from 38°C to 140°C.

**Figure 3:**
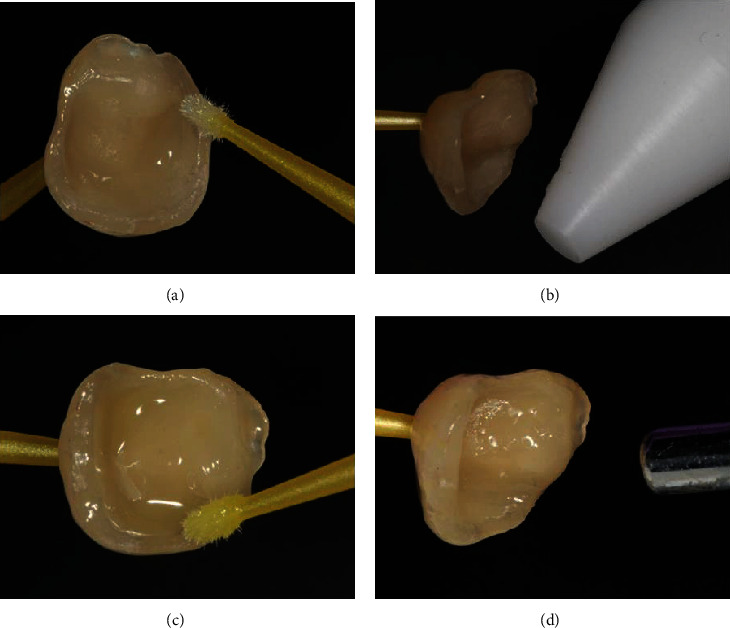
Bonding procedure: silane application (a); postsilanization heat treatment with custom-made device (b); bonding agent application (c); bonding agent solvent evaporation (d).

A constant 120°C airflow for 60 sec was applied, followed by dental adhesive OptiBond FL (Kerr Italia S.r.l., Scafati (SA), Italy). The steps are presented in Figure 3.

**Figure 4:**
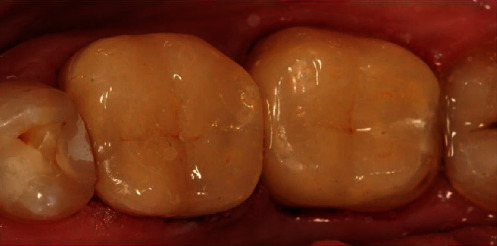
Postoperative view of the two overlays immediately after fixation and isolation removal.

The tooth structures were etched with 37.5% phosphoric acid (LOT 6775164, Gel Etchant, Kerr Italia S.r.l., Scafati (SA), Italy), and an OptiBond FL dental adhesive was applied. A flowable composite (Gaeneal Universal Flow, GC Corporation, Tokyo, Japan) was used for the permanent fixation. Light curing for 60 sec on each tooth surface was performed with Elipar™ DeepCure-S LED Curing Light (3М ESPE, St. Paul, USA). Occlusal contact check and adjustments were done after the final polymerization with 40 *μ*m articulating paper (Bausch Arti-Check, Dr. Jean Bausch GmbH & Co. KG, Koln, Germany). The final result is shown in Figure 4.

**Figure 5:**
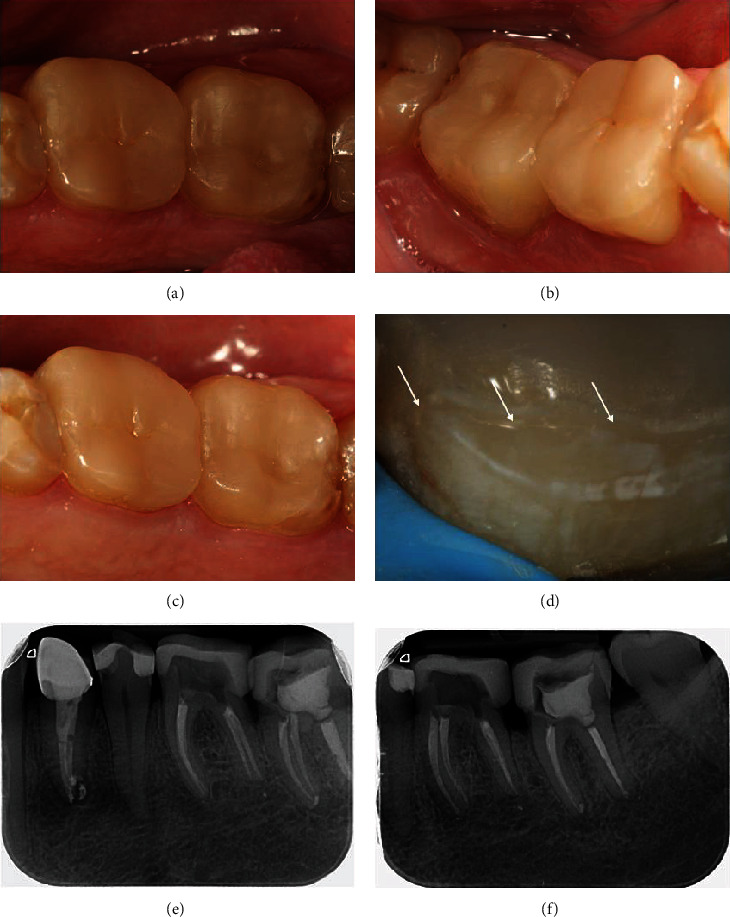
Four-year follow-up of the restorations: occlusal view (a); buccal view (b); lingual view (c); a close-up image of the overlay-tooth interface (d), white arrows pointing to the borders of the restorations; radiograph examination (e, f).

The FDI clinical criteria for the evaluation of direct and indirect restorations were used to evaluate the clinical appearance of the restorations after four years in use [22]. The appearance of the restorations could be assessed as clinically excellent/very good based on the aesthetic, functional, and biological properties. Lustre is comparable to that of enamel, and no surface staining, good color match, and ideal form preservation were observed. The absence of fractures, excellent marginal adaptation, normal contact points, and patient satisfaction correspond to clinical excellent/very good functional properties. The radiograph examination reveals a harmonious transition between the restorations and the tooth structures. We observed no hypersensitivity, no secondary or primary caries, very good periodontal response, healthy mucosa, and no general oral symptoms (Figure 5).

## 3. Discussion

This clinical result supports many in vitro studies regarding the resin-ceramic bond strength and durability obtained through postsilanization heat treatment.

The adhesive bonding procedure is one of the most critical steps determining the success and durability of LiS_2_ restorations. Proper tooth and ceramic preparation is mandatory. HF etching of the ceramic surface, followed by silane and dental adhesive application, is usually recommended for establishing a strong resin-ceramic bond. The HF etching dissolves partially the glassy phase of the silica-based ceramics, providing micromechanical retentions [23]. More undercuts and larger surface characterize etched ceramic compared to air-abraded ceramic; thus, a stronger resin-ceramic bond could be obtained [11, 24].

HF is a strong ceramic etching agent but potentially very harmful if it comes in contact with eyes, skin, or mouth tissues [16]. For this reason, some authors propose other etching agents, which are less effective, or to avoid the use of HF in the bonding procedure and use alternative protocols [25]. Despite the advantages of HF etching, it could weaken the material if the clinician exceeds the recommended etching time [26]. Overetching could also weaken the ceramic-composite bond strength [27]. HF promotes selective dissolution of the glassy phase of silica-based ceramics. The etching process includes several chemical reactions that lead to the deposition of insoluble silica-fluoride salts on the surface that negatively affects the bond. Thus, proper postetching cleaning of the ceramic is mandatory [28]. One could avoid these possible complications of the bonding procedure by excluding the HF etching in the bonding protocol. If so, to enhance the bond strength, proper postsilanization heat treatment combined with an appropriate microbraided ceramic surface is mandatory. To obtain a more durable resin-ceramic bond in the presented case, we applied postsilanization heat treatment at 120°C and avoided HF etching.

In addition to the micromechanical retentions, silane molecules can bond to both the functional groups of the silica phase of the ceramic and the resin material's organic matrix, thus providing chemical bonding between the two materials [29]. The efficacy of the chemical bonding depends on the type of the silane coupling agent, the material's application, and postsilanization treatment [30]. An interlayer is formed when a silane coupling agent is applied on the ceramic surface before resin application. Three different structures could be identified in the silane interlayer. The outermost layer comprises small oligomers, which could be easily eliminated by organic solvents and water at room temperature. The middle layer is formed by oligomers connected with stronger single siloxane bonds. In the inner layer, silane molecules form a network characterized by higher hydrolytic stability. Silane layer thickness should be as minimal as possible, and only the inner molecule layer is needed to obtain a strong and durable resin-ceramic bond. One of the most effective ways to obtain a highly condensed and stronger silane layer is postsilanization heat treatment [31].

Recommended temperatures vary among different authors. Fabianeli et al. and Roulet et al. propose 100°C heating temperature [17, 32]. Monticelli et al. use 38°C [33]. Hooshmand et al. tested three different temperatures—100°C (in oven), 80°C (boiled water), and 50°C (hot air) [34]. A recent study conducted a systematic comparison between different postsilanization heating temperatures. It showed the positive effect of higher temperatures on the bond strength between feldspathic ceramics and resin composite material [21]. The highest tensile bond strength was achieved among all groups when the bonding surface was treated with 120°C hot air. Since this was the most effective silane heat treatment method, we chose it appropriately in this clinical case. The 60 sec application of this temperature ensures a high condensation rate of the silane molecules; thus, a stronger chemical bond is achieved. It is harmless for both the patient and the clinician since the heating process is performed outside the mouth and the overlays are attached on a bonding stick.

Despite the very good outcome of the presented bonding method, there are some limitations. The follow-up period is relatively short (4 years). Different follow-up periods are reported in the literature—usually from 12 months to 15 years [2–4]. Thus, although the reported results are quite satisfactory, they should be considered preliminary. Since this case report presents only one patient and two overlays, it could not be accepted as scientific proof. Therefore, it could only be assumed as a promising result, and further randomized control trials should validate this approach and confirm the reliability of this method in vivo. Another limitation is the need for a specially designed hot air device, which is not supported by any manufacturer as far as we know.

## 4. Conclusion

The clinical appearance of the restorations could be defined as excellent/very good according to the FDI clinical criteria for the evaluation of direct and indirect restorations for the four-year follow-up period. Thus, it could be assumed that the bonding protocol used in this clinical case promotes a strong and durable ceramic-composite bond due to the combination of micromechanical retentions provided by air abrasion with AL_2_O_3_ and proper silane heat treatment. Although the results are promising, further studies should be conducted due to the limitations of this case report.
